# The care experience of people with diagnosed or suspected dementia living in prison: A case study approach

**DOI:** 10.1177/14713012251338873

**Published:** 2025-04-29

**Authors:** Rhoda MacRae, Natalie Chalmers, Debbie Tolson, James Taylor, Kirstin Anderson, Lindsay Thomson, Tom Russ

**Affiliations:** Alzheimer Scotland Centre for Policy and Practice, School of Health and Life Sciences, 6413 University of the West of Scotland, UK; School of Health in Social Science, 6413 University of the West of Scotland, UK; School of Applied Sciences, 3121 Edinburgh Napier University, UK; Alzheimer Scotland Centre for Policy and Practice, School of Health and Life Sciences, 6413 University of the West of Scotland, UK; School of Health and Life Sciences, 6413 University of the West of Scotland, UK; 3121 Edinburgh Napier University, UK; Centre for Clinical Brain Sciences, Division of Psychiatry, University of Edinburgh, UK; Department of Forensic Psychiatry, The State Hospitals Board for Scotland, UK; Centre for Clinical Brain Sciences, Division of Psychiatry, University of Edinburgh, UK

**Keywords:** dementia, prisons, health, care

## Abstract

Complex health and social care needs of people living in prison with diagnosed or suspected dementia is a growing concern for prisons and prison healthcare staff. The literature is replete with recommendations to better understand the health and social needs of this vulnerable population, to move beyond speculation towards actions to improve their health and well-being. Despite this, there is scant literature exploring the lived care experience of individuals being assessed for or diagnosed with dementia in prisons. The aim of this multi method qualitative study was to investigate how those with suspected dementia were identified, assessed and cared for in Scottish prisons. This article presents case study data from one phase of the larger study. Case studies were constructed from five semi-structured interviews with men with a diagnosed (*n* = 1) or suspected dementia (*n* = 4), four semi-structured interviews with staff the men nominated and data from the men’s health care records. The five men had multiple co-morbidities, three had significant mobility issues, two were in receipt of personal care and accommodated in accessible cells. Four of the five men exemplified previous descriptions of older prisoners in that they were socially and physically isolated, and reliant on support with everyday activities. A ‘case by case’ approach to referral, assessment and post diagnostic support was taken by staff who reported a complexity associated with meeting healthcare needs and access to specialist services and supports within a prison regime. This research provides unique and hither to seldom explored insight into the lived care experience of men living with a diagnosed or suspected dementia in prison. The findings have implications for how this marginalised vulnerable group are cared for in prison and on release.

## Background

The number of older people in prison is increasing globally (Maschi et al., 2021). In the UK the number of people in prison aged over 60 years has trebled in the last 20 years (Ministry of Justice, 2022). What constitutes ‘older’ varies in practice and research, usually between 50-60 years. Lower age thresholds are based on evidence that the health-related needs of prison populations are advanced by around 10 years relative to the general population (House of Commons Justice Committee Ageing prison population, 2020).

Physical and mental health care needs of older prisoners are complex and many (Fazel et al., 2001; Gilling McIntosh et al., 2023; Maschi et al., 2012). Older prisoners all experience extreme levels of stress (Stojkovic, 2007). This alongside social disadvantage, trauma, grief and loss, and childhood adversities all contribute to accelerated ageing (Fazel et al., 2006; Schnittker et al., 2012). Addressing health inequalities and providing equivalent health care in prison remains a challenge (Gilling McIntosh et al., 2023; McLintock & Sheard, 2024; Royal College of Nursing, 2016). Difficulties with recruitment, retention, and a lack of correlation between staff resource and the needs of a particular prison’s population often mean healthcare is crisis led (Mental Welfare Commission, 2022; Sheard et al., 2023). No prisons in the UK have hospital wards; if prisoners become acutely unwell they are transferred to hospital. In England and Wales, 40% of hospital outpatient appointments for prisoners were missed (Davies et al., 2021).

Many of the known risk factors for developing dementia are overrepresented in prison populations. Lower educational attainment, physical and mental health conditions, smoking, depression, diabetes, excessive alcohol consumption, traumatic brain injury, and high LDL cholesterol (Livingston et al., 2024). Additionally, those with a history of incarceration may have accelerated cognitive aging (Kuffel et al., 2022). The estimated prevalence of cognitive impairment and dementia in prison populations varies. In those over 50 years cognitive impairment estimations ranged from 1.3% (Forsyth et al., 2020) to 40% (Perez et al., 2021), 49% in those over 55 years (Ahalt et al., 2018). Likewise, estimates of dementia prevalence varies. In those over 50 years Forsyth et al., estimated 7.2%, (2020) in over 55 years estimations ranged from 9.1% (Baillargeon et al., 2023) to 20% (Ahalt et al., 2018). Few studies have undertaken comprehensive clinical assessments, nevertheless all suggest higher rates of cognitive impairment in prison populations than found in the community (Perez et al., 2021).

Forsyth et al. found 30% of prisons in England conducted routine cognitive screening (2020), although none of the four prisons in this study did so. Making a diagnosis in a population that often have several of the risk factors for dementia, lifelong disadvantages and intersecting mental and physical health problems is complex and requires specialist input (MacRae et al., 2024). Several studies have recommended dementia specific education is provided to prison staff to support identification and management (Brown, 2014; Gaston et al., 2022; Williams et al., 2012). However, education on ageing and dementia for staff working in prisons remains rare (Brooke et al., 2020; Gaston et al., 2022).

Prison staff have reported various challenges supporting people living with dementia in prison (Gaston, 2018). Specifically not knowing how to support this population within a prison regime and identifying the need for protocols or guidance to support care pathways (Brooke & Jackson, 2019). In the UK, the Royal College of Nursing produced a framework to promote good dementia care in prisons and tool to benchmark current practice (RCN, 2021). However, the extent to which this is used to support dementia care in prisons is unknown.

Previous studies have suggested that people living with dementia in prison may face victimisation social isolation, and depression (Dillon et al., 2019). It has also been argued that prison is an inappropriate setting for those with advanced dementia, who lack capacity, have specialist health, and care needs (Dillion et al., 2019). The provision of palliative and supportive care in prison is ill-defined, variable and beset with complications (Schaefer et al., 2022). Dementia specific palliative care adds another layer of complexity to supporting humane and dignified palliative and end of life care in prisons.

However there is a dearth of evidence about the lived care experience of individuals being assessed for or diagnosed with dementia in prisons. We know very little about the perspectives of these individuals in relation to their condition, their health and care needs, the challenges they face, their life in prison and how they see the future.

## Methods

### Aim

To describe the dementia lived and care experience in prison using a case study approach.

### Design

Findings from the larger study multi method qualitative study (Phases 1 and 3) can be found in MacRae et al. (2024). This paper focuses on phase 2, which used case study methodology to explore the lived care experience of men with a suspected (*n* = 4) or diagnosed dementia (*n* = 1) living in three prisons. The case studies were constructed from semi-structured interviews with five men, four individuals the men nominated, and data from the men’s health care records (see Table 1 for further details).

**Table 1.** Sample characteristics.

Pseudonym and case study number	Age	Cognitive screen results	Diagnosed dementia	Number of other health conditions	Number of prescribed medications	Receives personal care	Mobility issues	Nominated other
Davy – Case study 1	63	Not documented	No	4	7	No	No	Prison officer
Kenny- Case study 2	68	2022 MMSE 22/30 2022 MMSE 18/30	No	3	4	No	No	Prison officer
Fraser – Case study 3	67	Cognitive screen 2020 score not documented	Yes	8	10	Yes	Yes	Mental health nurse
George – Case study 4	79	2021 ACE-R 68/100	No	6	11	Yes	Yes	Mental health nurse
Robert – Case study 5	60	2018 MMSE 22/30 2020 MMSE 25/30 2021 MMSE 22/30 2022 ACE-R 52/100 2022 ACE-R 51/100	No	6	9	No	Yes	Mental health nurse

An Addenbrook’s Cognitive Examination-revised (ACE-R) score of 51/100 indicates significant cognitive impairment, an ACE-R score of 68/100 and a Mini Mental State Examination (MMSE) score of 18/30 indicates moderate cognitive impairment.

### Ethics approvals

The Scottish Prison Service Ethics and Access Committee gave approval access and ethical clearance in March 2021, the East of Scotland Research Ethics Committee gave the study favourable opinion on 06 May 2021 [21/ES/0034] and the research and development departments of the four Health Boards gave management approval between May and June 2021.

### Access and recruitment

Local access permissions were arranged with the Scottish Prison Service (SPS) governors and senior teams in the four sites. Both RM and NC conducted the interviews. Both underwent SPS orientation, SPS Personal Protection, Suicide awareness and management, and COVID-19 training. The NHS prison health board leads provided formal introductions to the Healthcare Centre Managers in the four prisons. During Phase 1, prison healthcare staff were asked to identify men with a diagnosed or suspected dementia. They were provided with the study inclusion and exclusion criteria, a participant information sheet, guidance to support staff help explain the study, and a ‘meet the researcher’ consent form. The inclusion criteria were the person was deemed safe to be interviewed; had capacity to provide informed consent; was willing to participate; was undergoing assessment for or diagnosed with dementia and had a least one month left to serve.

### Consent

Once consent was provided interviews were arranged. The researcher went through participant information with the men prior to interview, explaining that they could withdraw from the research at any time and participation, or non-participation, would have no bearing on their relationships with staff or care they received. After the men indicated their understanding, written consent was sought.

### Sampling and sample

A pragmatic sampling approach was used. Thirteen men were identified from health care records across the four prisons by healthcare staff. Five of the thirteen men did not meet the inclusion criteria as they were deemed to lack capacity and or were too ill to participate. Prison healthcare staff approached the remaining eight men – two declined and six consented to meet researchers. One man died before an interview was arranged. Five men were interviewed. Following interview, the researchers asked the men to nominate a significant other that could provide an insight into their lives. All men nominated staff and two of these men nominated the same member of staff. Four staff were interviewed. Following interview, all the men gave permission for prison health care staff to provide the research team with information from their health care records onto a questionnaire.

The age range was 60–79 years. All were on long term sentences (over four years). One had a clinical diagnosis of dementia. All had multiple co-morbidities, ranging from 3–8 conditions; all were prescribed multiple medications, ranging from 4–11 items. Two were in receipt of personal care to meet fundamental care needs such as washing and dressing. Three had significant mobility issues, either using a walking frame and or wheelchair to mobilise.

### Data collection and setting

Semi-structured audio-recorded interviews with the men were conducted either in a private room in the residential area or the healthcare centre depending on the availability of staff. The rooms had doors with windows providing sightlines to SPS staff who remained outside during interview. The interviews were 20–40 minutes duration. An indirect conversational approach was used to elicit what the men recollected about how their cognitive issues were identified, how they felt about support they received and plans or thoughts about release. A large-type card was used to rate on a scale of 1–10 how easy or difficult they were finding 14 activities of daily living. For the activities the men rated difficult, prompt questions were used to elicit more detail.

Semi-structured audio-recorded interviews with staff nominated by the men were conducted in a private room in their place of work. The interviews explored their understanding of about how the man had been identified as having cognitive impairment, their everyday life, enablers and barriers to providing care, interventions to promote well-being, issues relating to risk, progression and release, and dementia education provision and needs.

Questionnaire data were extracted from the men’s healthcare records by prison healthcare staff. Data included a small biographical section, a section for past and current medications, and 25 questions about medical issues.

The three data sources were combined to develop five case studies. Interview and questionnaire data were gathered between June 2021 and November 2022. All participants were given a pseudonym to preserve anonymity.

### Analysis

Interview data were transcribed, deidentified and uploaded to NVivo 12. Thematic Analysis (familiarisation, coding, generating initial themes, reviewing themes, defining and naming themes, writing up) was used to identify commonalities and patterns within and across the interview data (Braun & Clarke, 2006). NC generated codes and categories which RM reviewed, the three overarching themes: Identification and Assessment, Complex care needs and Dementia education were jointly generated.

## Findings

### Identification and assessment

Table 2 illustrates how the five men came to the attention of the Mental Health Team (MHT), the screening, assessment and referral processes and outcomes. ‘Not documented’ indicates the information was not provided via the returned questionnaires.

**Table 2.** Identification, screening and assessment.

Pseudonym and case study number	Age	Person and reason for referral to MHT	Date of referral to MHT	Length of time undergoing cognitive assessment	Other tests	Referred to old age psychiatry	Other referrals	Status at time of data collection
Davy - Case study 1	63	Self ‘pre-sentencing cognitive issues’	March 2022	2 months	Not documented	Not documented	Sessional GP	Initiating screening and assessment
Kenny – Case study 2	68	Social work for ‘cognitive issues’	August 2022	4 months	Bloods	Yes	Sessional GP	Awaiting response for old age psychiatry
Fraser – Case study 3	67	Not documented	Not documented	1 month	Not documented	Not documented	Not documented	Diagnosed dementia. No other data
George – Case study 4	79	Prison staff or ‘cognitive issues’	March 2021	15 months	CT scan x1	Yes, requested CT scan	Sessional forensic psychiatrist	Awaiting sessional psychiatrist to refer back to old age psychiatry
Robert – Case study 5	60	Prison staff for ‘communication, behaviour and memory issues’	May 2018	4 years	CT scan x4 Bloods x2 ECG	No	Neurology Sessional forensic psychiatrist	Awaiting referral to appropriate service

Davy (case study 1) had self-referred to healthcare because he was concerned about memory loss. George (case study 4) and Robert (case study 5) had been referred to healthcare by prison staff and Kenny (case study 2) by a social worker. These men had not approached prison or prison healthcare staff about any cognitive issues, two couldn’t recall undergoing any tests. Fraser (case study 3) had been clinically diagnosed with dementia; however we could not ascertain how he came to the attention of healthcare staff. Kenny, George and Robert were showing signs of moderate or significant cognitive impairment, for 4 months, 15 months and 4 years respectively, however they had not yet been diagnosed and were awaiting further assessment or referral.

Prison healthcare staff took a ‘case by case’ approach to someone presenting with cognitive impairment (MacRae et al., 2024). Usually, the mental health nurses carried out initial cognitive testing (see Table 1 for tests used). If further assessment was required, they would involve a sessional forensic psychiatrist, psychologist or GP and gather more in-depth health histories, request bloods and CT scans. The prison healthcare staff felt able to undertake assessments but felt that a specialist clinician, such as an old age psychiatrist needed to be involved in the diagnostic process. One prison had a newly established assessment and referral process agreed with their local health board old age psychiatry team.

“We didn’t have input until May this year [2022], when eventually we got a referral system in place, so we can now do onward referral to a memory clinic, for further assessment, we didn’t have that before. And they said that, if there is a concern due to a one-to-two-year history of cognitive changes, if we can provide a collateral history, an IQCODE^1^ to assess functional level, ACE III to assess cognition, routine bloods plus thyroid, calcium, B12 and folate, and the CT [brain scan] referral, once all the results are back, then we can refer to the psychiatry of old age. They will only see people over sixty-five. So if we’ve got anybody under, we have nowhere to take them”. Mental Health Nurse: Case study 4 and 5

Some memory clinics will accept referrals for people under 65 years and others have specialist memory clinics for under 65 years. All the prison healthcare teams remained unclear about where they would refer anyone under 65 years for specialist assessment.

Robert was 60 years old, and had been referred by prison staff several times over 4 years to healthcare for ‘communication, behaviour and memory issues’; they reported he could be irritable, hostile and aggressive. Robert had multiple co-morbidities and seemed to be presenting with a deteriorating and significant but fluctuating cognitive dysfunction, exemplifying the complexity of assessment and getting a differential diagnosis.

“I’ve discussed [Robert] with the consultant, they felt there were underlying physical health issues that needed further investigation. Because they felt that the man’s cognition functioning after getting the results of the CT scan and the hepatology ultrasound, wasn’t consistent with any kind of dementia. He felt it was consistent with a previous subdural haematoma, and that the primary care team should further investigate that. However, it doesn’t matter what the label is, or what the diagnosis is, he still has impaired cognition and functioning that we need to support him with”. Mental Health Nurse: Case study 4 and 5

Robert also described his fluctuating presentation; he thought he had performed well in the cognitive tests.

“Some days I’m alright and then the odd day I’m just puzzled, you know? It’s – nothing – nothing bothers me, know what I mean? That’s what it’s like. I’ve got no worries. Just sit and watch the telly, know what I mean?”. Robert: Case study 5

### Complex care needs

As noted in Table 1 all the men had several chronic health conditions (*n* = 3–8) and were on multiple medications (*n* = 4–11). Table 3 below shows how the men rated on a scale of 1(easy) and 10 (very difficult) they found 14 activities of daily living.

**Table 3.** Self-reported functional level for activities of daily living.

	Getting dressed	Going to the toilet	Taking a shower	Eating or drinking	Hearing	Seeing	Moving around	Work or recreation	Taking medication	Sleeping	Completing forms	Letter writing	Managing money	Phone calls
Davy - Case study 1	1	1	1	1	1	5 Glasses	1 Would get lost if not escorted	5	Supervised	5	10	1	1 in prison 5 outside	1
Kenny - Case study 2	2	1	1	1	1	5 Glasses	2 Only leaves room to go to toilet or eat	10	1	1	3	NA	1	2
Fraser - Case study 3	4	9	8 Personal care	3	1	6 Cataracts	9 Uses walking frame	5 Cannot work, recreation sometimes	Supervised	1	2	NA	9	2
George - Case study 4	10	10 Catheter	10 Personal care	5 Meals brought on tray	10 hearing aids	10 Glasses	10 Uses wheelchair	10 Cannot work	Supervised	1	10	NA	1	1
Robert - Case study 5	1	5	1	1	1	5 Glasses	5 Uses walking frame	10 cannot work	Supervised	1 with medication	2	NA	1	1

We can see that the men in receipt of personal care and with mobility issues self-reported more difficulty than the others. All had vision impairments, all reported moving or finding their way around either difficult or limited, and completing forms was very difficult for two.

Davy expressed less concern about his physical health than the other four men. Although on several medications, he was able to regularly participate in leisure and recreation activities. The three men with mobility issues were particularly concerned about their poor health and quality of life. Robert who used a walking frame, complained about the pain from his chronic leg ulcers which seeped and smelled, requiring dressing three times a week. He was concerned about a deterioration in his balance, after recently falling several times when going to the toilet at night. His declining eyesight and not having glasses to wear due to them being broken compounded his concerns about falling. George was in a wheelchair and had an indwelling catheter, was concerned about his leg ulcers not healing, but was more concerned about his impaired vision and hearing. He struggled to see and hear during the interview. He reported he was waiting to see the optician and his hearing aid had been in for repair for many months. Fraser used a walking frame, was also concerned about falling more frequently, and his worsening eyesight, he was waiting to be seen about his cataracts.

“I fall about, because I’ve no balance, and I keep telling them, and saying, “Look, I’ve no balance at all,” ken? “Oh right, right (name of person).” But nothing gets done. Honestly, it’s so irritating. It’s difficult for me to move about myself, you know? That’s terrible. I’m using that walker. It’s quite difficult, especially in the cell because there’s obstacles, you know? And you have to hang onto the sinks and the back of chairs and everything. I’m just no’ doing well in my cell”. Fraser: Case study 3

Fraser reported the nurses, officers and social workers deliberately withheld help and information and other prisoners were trying to steal his money. Although staff had referred him to an advocacy worker to talk about his concerns, he remained steadfast in his beliefs, which added another layer of complexity for Fraser’s care. Frasers’ notes recorded he was increasingly experiencing anxiety and low mood and he self-reported he was now reluctant to talk to anybody

“I had a couple of pals, but I’ve been shutting myself away. I just don’t want to know anybody. My mental health, it’s not good at all”. Fraser: Case study 3

The healthcare notes showed Robert and Davy had histories of anxiety and depression, George, Robert and Davy were prescribed medication for anxiety and depression. The four men who could not participate in work programmes needed assistance if they wanted to go outside or visit the library. They largely stayed in or near their cell, relied on other prisoners to remind or help them with ordering meals and personal items or clean their cell. They reported they had little social contact

“Not working, they won’t give me a job, health reasons. I keep myself to myself. If somebody comes in to see me I’ll talk away to them. But I’m usually sitting watching my telly, that’s what my life is now”. Robert: Case study 5.

“It’s good to get somebody to come and see you, because I’ve had no visitors, no nothing”. Kenny: Case study 2.

To try and better meet health and social care needs the four prisons tried to accommodate older men and those with health and social care needs together as much as possible. Prison managers also tried to allocate prison officers who were more experienced and or interested in working with this population to those areas. Kenny, Fraser and George were all accommodated in areas such as these. The nurse who managed the care of both George and Robert (who was accommodated in mainstream accommodation) explained some of the differences.

“One is getting better quality of care. Almost as good as you would get, I would think. Because the staff are now experienced in working with these guys, so we’ve had that sort of palliative care and high healthcare needs in for a good wee while now”. Mental Health nurse: Case study 4 and 5.

“[Robert’s] personal officer is very good and one of the other girls is also very good – but when they’re not there, it’s very different experience for him. His presentation fluctuates. Staff will report that he can be hostile and aggressive, non-compliant with his leg ulcer treatment, taking his dressings off, he plays about with the Entonox and won’t take it as he’s supposed to. They’ll have a spell where he’s angry and irritable all the time. Then we’ll see the man I saw yesterday, who was confused, reported things over and over again, and couldn’t remember things, but was pleasant, joking and laughing, and responding well to reorientation, guidance, redirection. And that is difficult stuff to grasp. “Is he at it? He’s at it.” You can understand why...with that fluctuating presentation”. Mental Health nurse: Case study 4 and 5.

Fraser and George were in receipt of personal care. Following an assessment of need by nursing staff, prison healthcare could request the prison service to contract external carers to provide personal care to individuals.

“I have carers helping me, yeah. But not with my memory, like. But the carers do help me, aye. Well they help us like if I’m having an accident, like, you know? I don’t like that much, them helping us in the shower. Because it’s embarrassing”. Fraser: Case study 3.

External carers focussed on personal care needs as identified by the nurses: showering, using the toilet, continence care, getting dressed, eating and drinking. These men also reported other prisoners supporting them.

“[other prisoners] they get me my meals. And they remind me things that I’ve got to do. I manage eating because they give me a table and bring it over and put it next to me”. George: Case study 4.

There were a very small number of accessible cells in each prison; these were slightly larger cells with a toilet. There were more men in need of these than there were cells available. Common adaptations included hospital bed and grab rails. Both Fraser and George were in accessible cells, George also had an airbed.

“We’re not a twenty-four-hour support system [prison healthcare], We do have carers, I think there’s fourteen, fifteen people that they care for, but not all on one block. I know the prison’s been good in trying to cohort people that have the most needs, but it’s not the best environment. The cells certainly are not adapted to anybody’s needs, whether somebody has dementia, or physical disabilities. It’s almost like we have to prioritise who gets what cell, based on who’s got the most need. Which is quite difficult, because you’re having to choose who’s needs are more than anybody else”. Mental Health nurse: Case study 3.

Health care staff felt that less staff resource was given to mental than physical health and this meant a disparity in care.

“So for physical health, I think we’re probably better at that. In our mental health team, we have seven nurses for nine hundred prisoners. So you can imagine with the level that some of these gentleman are at, they would benefit from a lot of support, which with all the good intentions in the world, we can’t offer”. Mental Health Nurse: Case study 3.

Based on the data from the returned questionnaires it appeared that none of the men had been referred to Occupational Therapy (OT), Speech and Language Therapy, or Physiotherapy. The mental health OTs in one healthcare team were only contracted to see people under 65 years. Another team had been trying to recruit a physiotherapist for many months without success. The healthcare staff wanted more specialist community input, specifically from mental health Occupational Therapists and Old age psychiatry teams. COVID-19 had resulted in many of the sessional services, such as audiology being stopped or reduced. By Spring 2022, these services had begun again, although the wait times for community services had increased during the intervening two years.

Davy and Kenny nominated prison officers to provide insights into their lives. Neither prison officer was aware that the men were undergoing cognitive assessments. Prison staff would only know about a person’s health conditions if the person told them, they had a communicable disease or health care staff had gained consent to share healthcare information.

“I’ve not been informed of any of the testing that’s been going on for [Davy]. One of my prisoners has just got diagnosed with diabetes, so I got informed about that, because obviously we to need to watch what he eats. Obviously with dementia, it’s more just watching him as a person and watch if he changes, or something’s a bit out of character. I’ve not had any experience with anybody with dementia. So even just giving me a heads-up, or a care plan, you know? Something, so I know what to look out for”. Prison officer: Case study 1

None of the men in the case studies were due for imminent liberation. Only one man talked about what they would do once they were released.

“When I go out I need to go into a care home. When I consider all the help I get in here, you know, I will need that outside. George: Case study 4

The staff reported arranging continuity of care was beset with complications. The nature and level the person’s health and social care needs, capacity and risk to the public all needed to be assessed. Assessing the risk to the public a person with dementia was difficult and complex. Staff reported that finding and securing alternative accommodation for people living with advanced dementia who lacked capacity, in receipt of personal care, with a criminal conviction (even if spent) was a long and challenging process as few alternatives were available. Compassionate release was being explored for one man.

“A lot of our gentlemen that we’ve got in, they’re in for a long time, they may never be liberated. We have our first compassionate liberation that we’re looking at, due to dementia”. Mental health nurse: case study 3.

### Dementia education

None of the staff from phase 2 had received any dementia education in their current role. The prison officers reported having had no dementia education during or since their training. One officer felt it would be useful to be able to recognise symptoms and understand how someone may present.

“I think giving staff more training in what to look out for, because you can obviously get the prisoners that are quite forgetful, et cetera, but just making personal officers aware that prisoners are going through testing for it. We need to know these things, it’s our duty of care to look after them, so we need to be able to look out, so that if something was to change with them, whether it’s like, “That’s a bit weird,” we know that there’s a reason for that”. Prison officer: Case study 1

Both the mental health nurses had post-qualification experience of working with older people and people living with dementia, however, felt that the increasing numbers of older prisoners, many with complex care needs was not yet recognised as something that needed specialist staffing resource or education.

“We’re going to get more and more older people coming in that are going to need assistance from us. So any training, from the basics right up to more complex. Most of us are lacking that. We have nurses that specialise in ADHD and ASD. We consider dementia a speciality as well. But it’s something that has not been recognised, when people are factoring in funding and what staffing we need for it”. Mental health nurse: Case study 3

## Discussion

Five of the thirteen men identified by healthcare staff were deemed not to have capacity due to their advanced dementia. Of the five that participated, three were showing signs of moderate or significant cognitive impairment and had four had complex care needs that often accompanies moderate or advanced dementia. This suggests that people with a suspected dementia are not being identified, assessed or diagnosed until they are significantly impaired. This supports previous findings that found 3% and 15% of those who screened positive for dementia had a diagnosis recorded (Baillargeon et al., 2023; Forsyth et al., 2020). The lack of routine cognitive screening in prisons places a reliance on staff, particularly prison staff to recognise signs of cognitive impairment. In a prison environment the opportunities to notice early signs of dementia such as struggling to manage money, shopping and driving will be fewer (Simmons et al., 2011). Missing out on timely diagnosis negates potential benefits such as early treatment, reducing anxiety about symptoms, access to resources and supports, future planning and perhaps particularly in this setting, symptoms not being seen as challenging behaviour.

Four of the men reported struggling with all activities of daily living such as getting dressed, personal hygiene, mobilising, wayfinding, making decisions and socialising, impacting negatively on their quality of life. Poor quality of life and interactions between dementia and co-morbidities further increase morbidity and mortality (Subramaniam, 2019). The four men appeared apathetic about living in poor health in prison, and their future. Three were prescribed medication for depression, a common overlapping symptom with dementia (Dillion et al., 2019).

SPS tried to house older men with care needs together so they could facilitate the provision of care more efficiently and staff the area more effectively. External carers supported personal care rather than social care which would include supporting wellbeing and opportunities to participate. Implementing a narrower definition of social care impacts on how need is perceived, self-reported and provided (Levy et al., 2018; MacRae et al., 2024). Housing older men with care needs together seemed to support the provision of personal care, although the cramped spaces with fixed fittings made it difficult for both those giving and receiving personal care. However it appeared that the wider social care needs of the men went unmet. Our findings support previous research on older prisoners which asserted that the combination of isolated periods, with no purposeful activity, where mobility is not facilitated leaves them prone to loneliness, isolation, anxiety and depression (Baidawi et al., 2016). One prison had successfully implemented some environmental modifications enabling an individual to manage diet and fluids more independently. However, none of the men we spoke to had experience of this despite three of them struggling to mobilise and way find, particularly at night alone in their cell.

The prison setting also constrained the provision of healthcare (Trotter & Baidawi, 2015). Some reported missed hospital appointments and waiting months for opticians or audiologists’ appointments. These delays may have been COVID 19 related, however the men perceived them to be due to failures in communication between prison and healthcare staff or lack of staff. Both prison officers were not aware of concerns about Davy and Kenny’s cognition. This constrained officers’ ability to respond appropriately to the needs of these men.

Only one man seemed able to consider what life on release may look like, at 79 years old with deteriorating health, for him, 24 hour care seemed to be the only possibility. However, there are very real challenges finding suitable accommodation for prisoners (Dillon et al., 2019). The ethics of keeping men with advanced dementia incarcerated, who may not understand where they are or why they are there, has been raised previously (Fazel et al., 2002). If release is not an option, those with advanced dementia will require palliative and end of life care. Dementia specific palliation, although advocated (van der Steen et al., 2014) is seldom implemented in the community (Brennan et al., 2023) and end of life care for people in prison is inadequate (Hospice UK, 2021). Yet, there will be people with advanced dementia who will require palliative and end of life care in prison.

The calls to provide dementia education to staff are not new. Prison healthcare staff, prison officers and those living alongside older prisoners need to be educationally supported to recognise signs and symptoms, understand how to provide trauma informed, relationship centred dementia care and take a compassionate approach to a person’s health and social care needs throughout the trajectory of the illness. In effect, be supported and resourced to implement dementia care frameworks (RCN, 2021) and pathways of care (MacRae et al., 2024)

### Merits and limitations

To the best of our knowledge, this is first study to explore the lived experience and gather the health histories of people living with a diagnosed or suspected dementia in Scottish prisons. All thirteen men identified in the four prisons were over 60 years, and four of the five were awaiting diagnosis, so our findings are limited in those respects. Four of the five men had signs and symptoms of significant cognitive impairment which likely affected their recall. However, by triangulating the data from staff, healthcare records and the men we were able to build up a picture of their lived care experience. We attempted to include people with advanced dementia; however, we were unable to gain ethical approval to do so. This has been described elsewhere as epistemic injustice and limits the voice of people living with dementia (Halonen et al., 2024). The data were gathered between April and November 2022, following a six-month data collection pause due to the impact of COVID-19 on prison and prison healthcare staffing levels. Although the prison officers felt they did not know the men well, and one of the returned questionnaires contained scant detail about one man’s health history, the approach nonetheless provided evidence critical to understanding about the lived care experience and health profiles of those with a diagnosed or suspected dementia in prison.

### Implications and conclusions

Our sample indicated that people with a suspected dementia were being identified only once they were showing signs of significant impairment. Identification was largely reliant on a workforce with no dementia education. There is an argument for introducing routine cognitive screening for this population who are vulnerable to accelerated ageing and poor cognitive health. Pathways for pre and post diagnostic dementia care been created, it will be important for local prison healthcare teams to be aware of and supported to use these (Forsyth et al., 2020; MacRae et al., 2024). If implemented, this would enable people to be identified, assessed and diagnosed sooner. Implementation of the model and co-produced pre and post diagnostic pathway together would promote an integrated approach to care provision for this group (MacRae et al., 2024). There is an educational need amongst staff working in prison to understand more about how to support healthy ageing, and the interactions between dementia and co-morbidities to support a better quality of life amongst this increasing population. There is a plethora of dementia education available and accessible to the health and social care workforce, it is important that prison and prison healthcare staff are made aware of how to access this and that it forms part of their training, induction and continuing professional development. However, it is also likely that a targeted approach to dementia education would need to be facilitated to ensure the educational needs of staff described above were met.

External carers provided personal care; however, their role could be extended to include meeting wider social care needs such as socialising and purposeful activity for this population with guidance from allied health professionals (AHPs). The numbers, types, and scope of AHPs in healthcare teams varied greatly, however this professional group are well placed to support healthy ageing with their knowledge of physical, environmental, and dietary modifications, purposeful and physical activity.

There have been developments in the provision of palliative and end of life care in prison, however this remains patchy and largely inadequate (Hospice UK, 2021). There is a need for palliative and end of life care policies, approaches, and education to include dementia specific palliation both in the community and prison. Compassionate release provisions are often underutilised in practice due to restrictive criteria including severe dementia (Kaushik & Currin-McCulloch, 2023) so it is likely that this population will either die in prison or be released. Therefore it seems important to identify care homes and services that are willing to provide care to this population to support timely release and continuity of care.

Future research could assess the feasibility and acceptability of pathways where they have been implemented, exploring the factors affecting implementation to understand the facilitators and barriers to success. Further research into the lived experiences of people living with dementia in prison, particularly those who are deemed not to have capacity and living with advanced dementia is needed to understand how risk is assessed, compassionate release considered, and palliative care provided.

## Data Availability

The datasets used and or analysed during the current study are available from the corresponding author on reasonable request. *

## Note

1. The IQCODE is a brief screening questionnaire to determine whether day-to-day functioning has declined over time, it can be completed by someone that supports the person. It is usually used in combination with cognitive tests to improve detection of dementia.
